# Correction: Learning-induced ribosomal RNA is required for memory consolidation in mice—Evidence of differentially expressed rRNA variants in learning and memory

**DOI:** 10.1371/journal.pone.0207142

**Published:** 2018-11-05

**Authors:** The PLOS ONE Staff

## Notice of republication

This article was republished on October 26, 2018 to correct for an error in the title that was introduced during the typesetting process. The publisher apologizes for the error. Please download this article again to view the correct version. The originally published, uncorrected article and the republished, corrected article are provided here for reference.

## Supporting information

S1 File Originally published, uncorrected article. (PDF)

S2 File Republished, corrected article. (PDF)
