# Supplementary figures and images for: Learning-induced ribosomal RNA is required for memory consolidation in mice—Evidence of differentially expressed rRNA variants in learning and memory
Source: PLoS One. 2018 Oct 3;13(10):e0203374. doi: 10.1371/journal.pone.0203374 (PMC6169870; doi:10.1371/journal.pone.0203374)

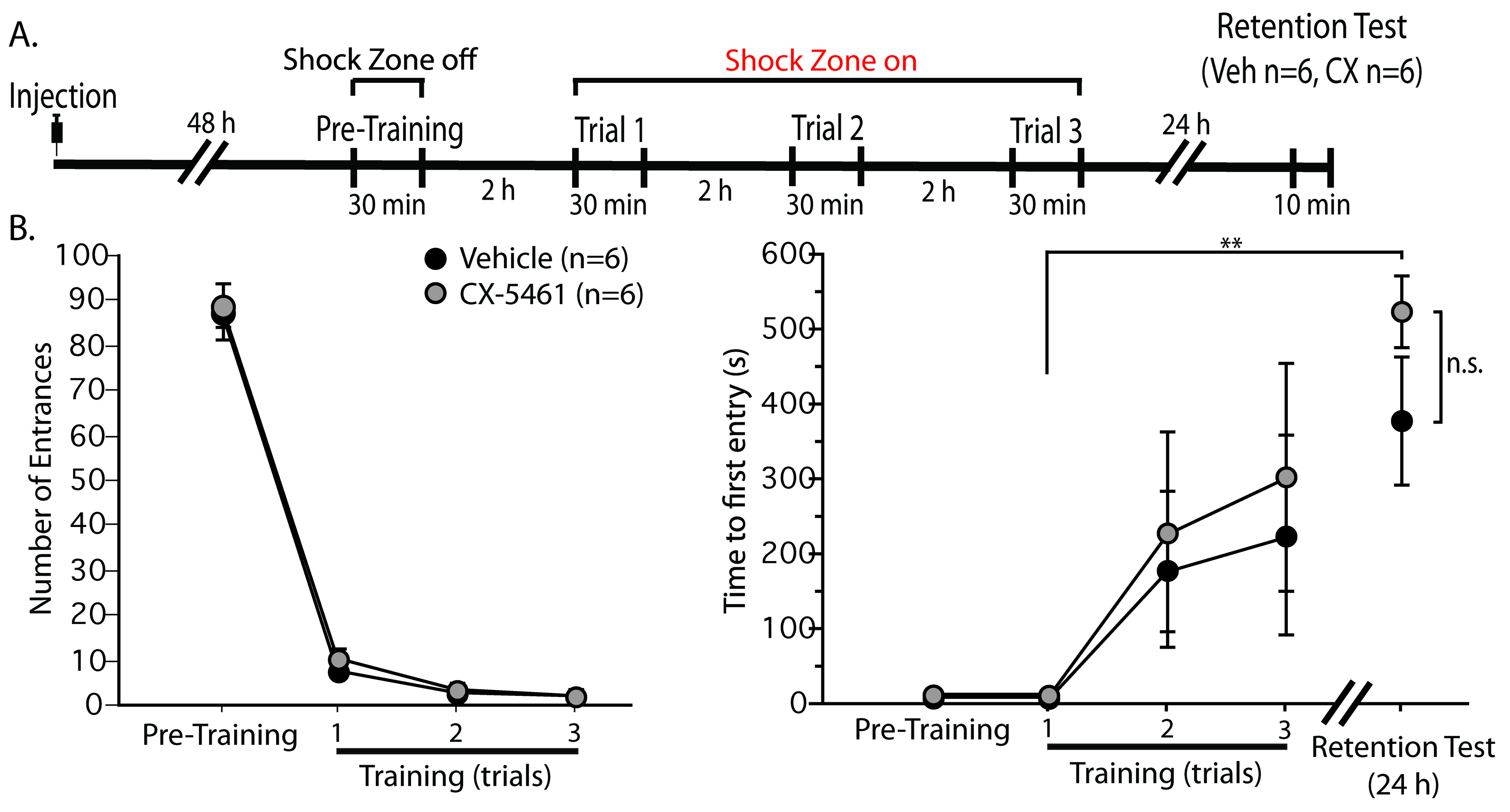

Supplement: S1 Fig — A) Timeline of the training protocol. Animals were injected with CX-5461 or vehicle 48 h before commencement of training. B) Comparison of learning and memory between APA trained mice who received intracranial injection of vehicle (black circles) and CX-5461 (grey circles). Left, Number of entrances during the 30 min training trials. Right, Time to first entry into the shock zone during each training trial and the 24 h retention test. Both CX-5461 and vehicle injected animals significantly reduced the number of entrances during training, and increased their time to first entrance indicating that they learned the location of the shock zone. A significant increase in the time to first entry during the retention test indicates that both groups exhibited memory of the shock zone 24 h after training. No significant differences in the memory retention test were observed between the two groups indicating that the group treated with CX-5461 does not lose the ability to consolidate memory. (TIF) [file pone.0203374.s001.tif]

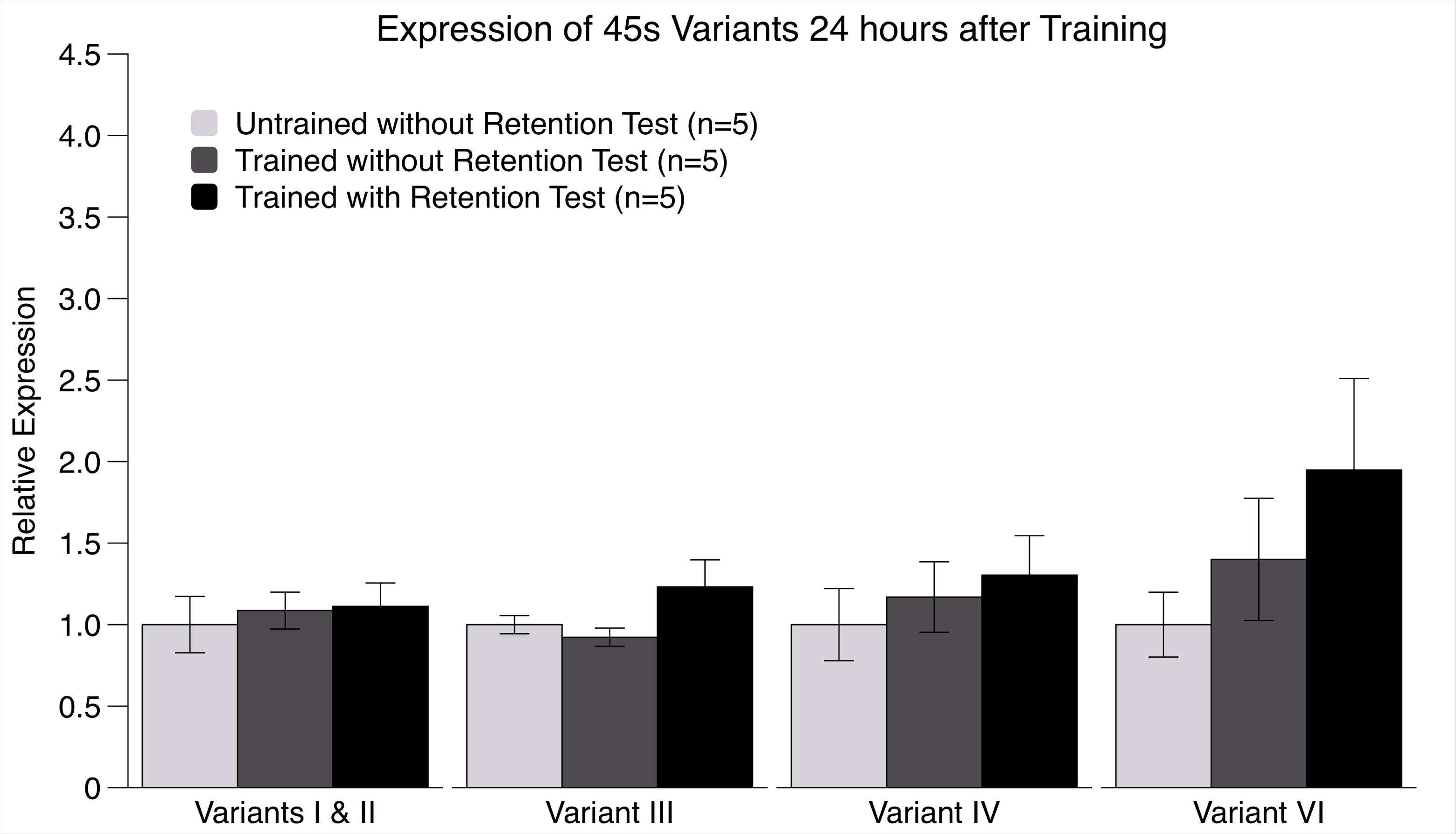

Supplement: S2 Fig — Real-Time qPCR analysis demonstrates that none of the five known hippocampal rRNA variants are significantly upregulated 24 h after the third APA training trial, whether or not an animal received a retention test. [Significance analyzed by ANOVA. All p values > 0.14]. (TIF) [file pone.0203374.s002.tif]

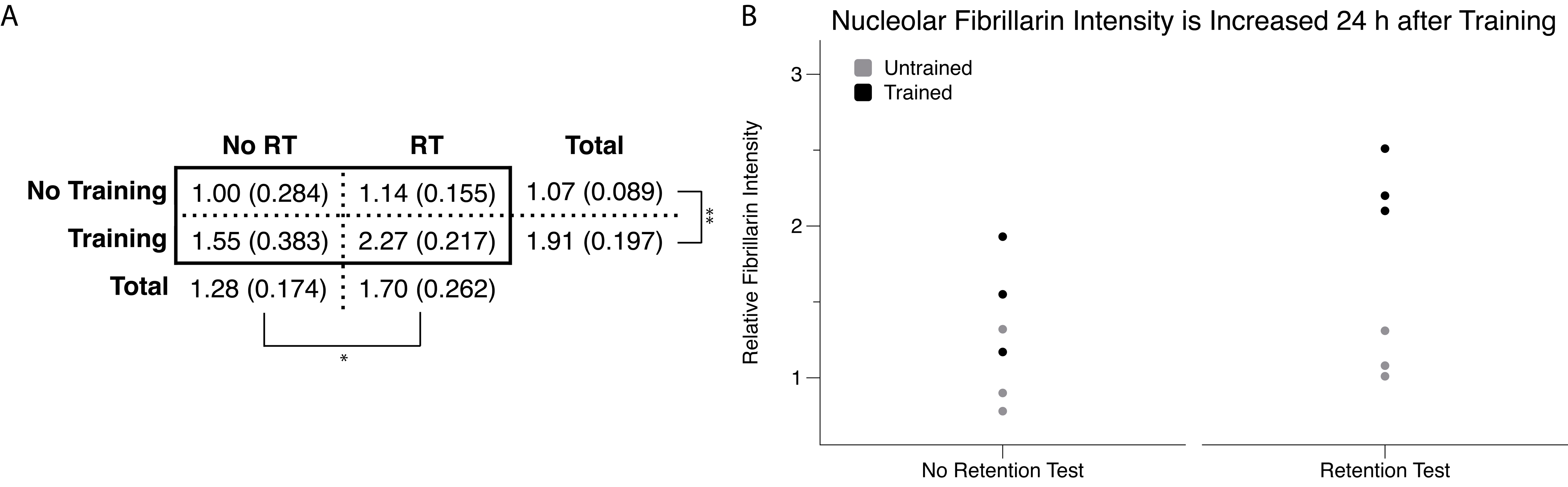

Supplement: S3 Fig — A) Table showing mean (and standard deviation) for each of 4 groups (n = 3/group) (trained and untrained with and without RT), as well as total for each condition. A two-way ANOVA found increased fibrillarin staining in response to training (F1,8 = 28.484, p = .001) and RT (F1,8 = 7.351, p = .027), but not the interaction of the two Training*RT (F1,8 = 3.405, p = .102). B) Graphical representation of the same data showing an effect of RT (right panel compared to left) and training (black dots compared to grey). Each dot represents the average fibrillarin intensity of an animal. (TIF) [file pone.0203374.s003.tif]
