# Supplementary material for: Learning-induced ribosomal RNA is required for memory consolidation in mice—Evidence of differentially expressed rRNA variants in learning and memory
Source: PLoS One. 2018 Oct 3;13(10):e0203374. doi: 10.1371/journal.pone.0203374 (PMC6169870; doi:10.1371/journal.pone.0203374)
Supplement: S1 File — Description of the methods used to produce the data presented in S1–S3 Figs. (DOCX) [file pone.0203374.s004.docx]

**Supporting information methods**

**S1 Fig methods**

Stereotaxic intrahippocampal cannulation surgery and drug injection were done as described in the main manuscript for figure 3, with the exception that the drug was administered 48 h before training.

**S2 Fig methods**

RNA purification, reverse transcription, and qPCR analysis were all performed as described in the manuscript. Additionally, the behavior protocol was the same as described for figure 5. Since there are no differences between Untrained with or without retention test, only the latter is presented in this figure as a control.

**S3 Fig methods**

Immunohistochemistry is described in the manuscript. The data are derived from four groups: Untrained, no retention test; Untrained with retention test; Trained, no retention test; and Trained with retention (n=3 animals/group). Mice received the Retention test 24 h after the last exposure to the APA arena; after which, they were perfused for IHC. Animals that did not receive a retention test were perfused in close propinquity to their retention tested counterparts.

All images were analyzed using Olympus Fluoview ver. 2.1a (FV10-ASW version 02.01.01.04). The region analyzed was the CA1 region of the dorsal hippocampus. Pyramidal cells to be quantified were determined by nuclear DAPI staining without looking at the fibrillarin staining--nuclear regions of interest (ROIs). For each animal, more than 150 cells were scored. Nucleolar ROIs were determined by the extent of fibrillarin staining within the nuclear ROIs. The average fibrillarin intensity within the ROI was quantified. Intensity measurements were taken on a standard 0-255 pixel intensity scale; the results were averaged and control normalized to one.

*Statistics for figure 3*: A two-way ANOVA was performed using training and retention test as parameters. The Levene test was run to check equality of variances (F = 6.17; p= .623). We found a significant effect of training (F_1,8_ = 28.484, p = .001) and retention test (F_1,8_ = 7.351, p = .027) on fibrillarin staining. No significant interaction (Training*RT) was found (F_1,8_ = 3.405, p = .102).
