# Supplementary material for: Correction: Learning-induced ribosomal RNA is required for memory consolidation in mice—Evidence of differentially expressed rRNA variants in learning and memory
Source: PLoS One. 2018 Nov 5;13(11):e0207142. doi: 10.1371/journal.pone.0207142 (PMC6218089; doi:10.1371/journal.pone.0207142)
Supplement: S1 File — (PDF) [file pone.0207142.s001.pdf]

PLOS | ONE

RESEARCH ARTICLE

Learning-induced ribosomal RNA is required for memory consolidation in mice—Evidence of differentially ex-pressed rRNA variants in learning and memory

Kim D. Allen[1,2☯], Matthew J. Regier[1,3☯], Changchi Hsieh[4], Panayiotis Tsokas[4,5], Maya Barnard[1], Shwetha Phatarpekar[1,3], Jason Wolk[6], Todd C. Sacktor[4,5,7,8], André A. Fenton[4,8,9], A. Iván Hernández[1,8]*

1 Department of Pathology, State University of New York, Downstate Medical Center, Brooklyn, New York, United States of America, **2** Department of Biology, School of Science, Health and Technology, City University of New York, Medgar Evers College, Brooklyn, New York, United States of America, **3** Graduate Program in Neural and Behavioral Science, State University of New York, Downstate Medical Center, Brooklyn, New York, United States of America, **4** Department of Physiology and Pharmacology, State University of New York, Downstate Medical Center, Brooklyn, New York, United States of America, **5** Department of Anesthesiology, State University of New York, Downstate Medical Center, Brooklyn, New York, United States of America, **6** Department of Physics, New York University, New York, New York, United States of America, **7** Department of Neurology, State University of New York, Downstate Medical Center, Brooklyn, New York, United States of America, **8** The Robert F. Furchgott Center for Neural and Behavioral Science, State University of New York, Downstate Medical Center, Brooklyn, New York, United States of America, **9** Center for Neural Science, New York University, New York, New York, United States of America

☯ These authors contributed equally to this work.
* ivan.hernandez@downstate.edu

Check for updates

OPEN ACCESS

Citation: Allen KD, Regier MJ, Hsieh C, Tsokas P, Barnard M, Phatarpekar S, et al. (2018) Learning-induced ribosomal RNA is required for memory consolidation in mice—Evidence of differentially ex-pressed rRNA variants in learning and memory. PLoS ONE 13(10): e0203374. https://doi.org/10.1371/journal.pone.0203374

Editor: Michal Hetman, University of Louisville, UNITED STATES

Received: November 10, 2017

Accepted: August 20, 2018

Published: October 3, 2018

Copyright: © 2018 Allen et al. This is an open access article distributed under the terms of the Creative Commons Attribution License, which permits unrestricted use, distribution, and reproduction in any medium, provided the original author and source are credited.

Data Availability Statement: All relevant data are within the paper and its Supporting Information files.

Funding: The authors received no specific funding for this work.

Competing interests: The authors have declared that no competing interests exist.

Abstract

The transition from short-term to long-term forms of synaptic plasticity requires protein synthesis and new gene expression. Most efforts to understand experience-induced changes in neuronal gene expression have focused on the transcription products of RNA polymerase II—primarily mRNAs and the proteins they encode. We recently showed that nucleolar integrity and activity-dependent ribosomal RNA (rRNA) synthesis are essential for the maintenance of hippocampal long-term potentiation (LTP). Consequently, the synaptic plasticity and memory hypothesis predicts that nucleolar integrity and activity dependent rRNA synthesis would be required for Long-term memory (LTM). We tested this prediction using the hippocampus-dependent, Active Place Avoidance (APA) spatial memory task and found that training induces *de novo* rRNA synthesis in mouse dorsal hippocampus. This learning-induced increase in nucleolar activity and rRNA synthesis persists at least 24 h after training. In addition, intra-hippocampal injection of the Pol I specific inhibitor, CX-5461 prior to training, revealed that *de novo* rRNA synthesis is required for 24 h memory, but not for learning. Using qPCR to assess activity-dependent changes in gene expression, we found that of seven known rRNA expression variants (v-rRNAs), only one, v-rRNA IV, is significantly upregulated right after training. These data indicate that learning induced v-rRNAs are crucial for LTM, and constitute the first evidence that differential rRNA gene expression plays a role in memory.

Introduction

In 1950, Katz and Halstead first proposed that memory formation required new protein synthesis) [1] —a hypothesis that was not tested until decades later [2–4]. It is now well accepted that memory consolidation requires new transcription and new activity-dependent protein synthesis [5, 6]. Most efforts to understand experience-induced changes in neuronal gene expression have focused on the transcription products of RNA polymerase II (Pol II)—primarily mRNAs and the proteins they encode [6, 7]. While there has been significant progress in identifying the Pol II dependent transcripts (primarily mRNAs) required for early phase long-term synaptic plasticity, the gene products responsible for late-phase and maintenance remain elusive. In contrast, the transcription products of RNA polymerase I (Pol I), responsible for producing non-(protein) coding ribosomal RNA, have been left unexplored despite the fact that Pol I transcription constitute more than 50% of nascent RNA synthesis in a cell (reviewed by [8, 9]).

In eukaryotes, rDNA exists as multiple tandem repeats organized into one or more non-membrane bound organelles— subnuclear compartments called nucleoli [10]. Each transcription unit generates a 45S precursor rRNA that is then edited, modified and assembled into ribosomal subunits. Nucleoli form through the act of producing ribosomes [11]. Each stage of ribosome biogenesis corresponds to a structural feature of the nucleolus, such that ultra-structural features, (e.g., the size and shape of the nucleolus), directly relate to nucleolar function and the production of ribosomes (reviewed by [12]).

Our prior work in *Aplysia* provided the first evidence that neuronal stimulation evokes poly ADP ribose polymerase-1 (PARP-1) dependent *de novo* rRNA synthesis [13]. Recently, by examining hippocampal long-term potentiation (LTP), widely considered a physiological substrate of memory [14], we found that nucleolar integrity—and specifically, *de novo* rRNA synthesis, is required for the maintenance of LTP [15]. This plasticity-dependent rRNA expression is activated by the PKA-ERK pathway, which in turn activates nucleolar PARP-1 to induce Pol I rRNA synthesis required for the formation of new ribosomes. Since new Pol I transcription is necessary for late-phase LTP (L-LTP), we hypothesized that it would also be important for LTM. We tested this prediction in mice using the hippocampal dependent Active Place Avoidance (APA) task [16, 17] and found, as anticipated, that new rRNA synthesis is required for 24 h memory. Furthermore, we show for the first time that rRNAs are differentially regulated in learning and memory. We discuss the important implications of these findings to the memory impairment characteristic of Alzheimer's Disease.

Materials and methods

Animals: B57/BL6 Mice (male, 2-4 months)

All procedures comply with the Public Health Service Policy on Humane Care and Use of Laboratory Animals and were approved by the State University of New York, Downstate Medical Center Animal Care and Use Committee. All efforts were made to minimize the number of animals used and their suffering. For qPCR studies, mice were euthanized by decapitation following deep anesthesia with 5% vaporized isoflurane in oxygen (100%). After decapitation, the brain was rapidly removed and placed into ice cold dissection artificial cerebrospinal fluid (dACSF, containing in mM: 125 NaCl, 2.5 KCl, 7 MgSO4, 0.5 CaCl2, 25 NaHCO3, 1.25 NaH2PO4 and 25 Glucose) oxygenated with a 95%O2/5%CO2 mixture (ACSF pH 7.3). The dorsal hippocampus was dissected out and processed for qPCR studies. For immunohistochemistry studies, mice were perfused through the aorta for 3 min with ice cold 4% PFA in PBS, pH 7.4, and the brains were fixed overnight in the same buffer at 4°C.

Molecular biology

Detailed descriptions of molecular techniques such as RNA isolation and qPCR were previously published [15]. RNA isolation: Total RNA was isolated from dorsal hippocampi using TRIzol reagent (Invitrogen) according to the manufacturer's instructions. Reverse transcription: cDNA was generated from RNA samples using the Superscript III First-Strand Synthesis protocol for random hexamers (Invitrogen).

Quantitative Real-Time PCR (qPCR)

Comparative Ct qPCR was performed using SYBR-Green RT-PCR Master Mix detection reagent (Applied Biosystems) and Stratagene's Mx3000P Real-Time PCR system. Each 20 μl qPCR reaction contained 2.5 ng of cDNA. The final primer concentration was 0.5 μM. The thermocycling conditions were as follows: 50˚C for 5 min (1 cycle), 95˚C for 10 min (1 cycle), 60˚C for 1 min (1 cycle), and 95˚C for 15 s followed by 60˚C for 1 min (40 cycles). At the end of the protocol, a dissociation curve (start temperature 55˚C) was performed to assess the specificity of amplification. In order to quantify stimulation-dependent changes in target gene expression, samples were normalized to the housekeeping gene *GAPDH*, previously found to be constitutively abundant in the hippocampus [18]. The primers used for mouse *GAPDH* were: *m-gapdh* F 5′-TTGTGATGGGTGTGAACCACGAGA-3′ and *m-gapdh* R 5′-GAGCCC TTCCACAATGCCAAAGTT-3′. Newly synthesized precursor rRNA was quantified using primers specific for a region of heterogeneous 45S pre-rRNA (ht rRNA) between the internal transcribed spacer 2 (ITS2) and the 28S rRNA.

45S (ht rRNA) primers: *ht rRNA*-F 5′-GCCGGGTGCCGTCTCTTT-3′ and *ht rRNA*-R 5′-TA TGCTTAAATTCAGCGGGTCGCC-3′. The IEGs *c-jun* and *c-fos* were employed as Pol II-dependent, plasticity-induced positive controls. Primers for mouse IEGs were: *c-jun* F 5′-G AACTGCATAGCCAGAACACGCTT-3′ and *c-jun* R 5′- TGAAGTTGCTGAGGTTGGCGTAGA-3′; *c-fos*-F 5′ ATCGGCAGAAGGGGCAAAGTAG-3′ and *c-fos* R 5′ GCAACGCAGACTTCTC ATCTTCAAG-3′. Primers to detect *v-rRNAs*: *I,II* F 5′-TCCCGGTCTTTCTTCCAC-3′; *v-rRNA I,II*-R 5′-CATGAA CACTTGGACACCA-3′; *v-rRNA III* F 5′-CCGAGTACTTCTC CTGTCTG-3′; *v-rRNA III*-R 5′-CAAGACAGTTACGGATACGG-3′; *v-rRNA IV*-F 5′-AA GTTTCTCGAGAGACTCATG-3′; *v-rRNA IV*-R 5′-TTCTCTTCCAAGGGCATTC-3′; *v-rRNA VI*-F 5′-CAGAATGCCCTTGGAAGG-3′; and *v-rRNA VI*-R 5′-CACACAGGGAAACCAGA AG-3′. Each new primer was tested for specificity and priming efficiency (Applied Biosystems *GeneAmp 5700 Sequence Detection System).*

Immunohistochemistry

After 24 h of fixation in 4% PFA, 40 μm coronal brain slices were made using a Leica VT 12005 vibratome (Leica Biosystems) and transferred to multi-well tissue culture plates filled with phosphate buffered saline (PBS). To improve antibody penetration, the slices were treated with 1% SDS detergent in PBS for 10 min and rinsed with PBS 4X (10 min). From there, slices were transferred to quenching solution (0.2% glycine in PBS) for 10 min and rinsed with PBS 3X for 10 min. To expose the fibrillarin epitope, the slices were placed into trays filled with 30 mM sodium citrate which were then incubated for 30 min in a water bath preheated to 80˚C. They remained in the solution while being allowed to return to room temperature. The slices were then rinsed with PBS 3X (5 min) and incubated overnight in blocking solution (2% Normal Goat Serum, NGS, in PBS). After blocking, slices were incubated overnight in rabbit anti-fibrillarin polyclonal antibody (1:500; cat# ab5821, Abcam) diluted in blocking solution or incubated overnight in blocking solution alone (no primary control, NP). Next, slices were rinsed with PBS 4X (15 min each) and incubated overnight in secondary antibody (Goat Anti-

Rabbit Alexafluor 488; 1:200) in blocking solution. Lastly, the slices were rinsed with PBS 4X (10 min each) before being transferred to slides and mounted with antifade (DAPI Fluoromount-G; Southern Biotech).

Active Place Avoidance (APA) training

See references [16] and [19] for detailed descriptions of the hippocampus-dependent APA task. Briefly, mice on a slowly (1 rpm) rotating 40 cm diameter circular arena must avoid a nonrotating 60° unmarked sector (the shock zone). The rotation brings the animal into the shock zone unless it learns to avoid the zone using distal spatial cues set outside the arena. A constant current foot-shock (60 Hz, 500 ms) was delivered whenever the mouse entered the shock for at least 500 ms. The shock was repeated each 1500 ms until the mouse left the shock zone. The shock amplitude was 0.2 mA, the minimum required to elicit an escape response. We used a one-day training protocol in which the animals received a 30 min pre-training session followed by three 30 min training trials. Each 30 min session (pre-training and training trials) was separated by 2 h. The position data were analyzed offline (TrackAnalysis, Bio-Signal Group) and used to extract measures that assess learning and memory. The number of entrances into the shock zone decreases with training and estimates learning, the time to first enter the shock zone estimates memory. One-day long-term memory was assessed with the shock off, by measuring how well the animal avoided the former location of shock during a 10 min retention test administered 24 h after the third training trial. In the first series of experiments (Fig 1), three training groups were compared: 1) "Trained" animals received APA training that challenged them to learn the location of shock using distal cues. 2) "Yoked" animals had the identical physical experience but could not learn the location of shock because they received shocks in the same time sequence as a counterpart animal in the Trained group so that the shock was delivered randomly with respect to the mouse's location. 3) "Untrained" animals had the identical training experience with the exception of shock, which was never experienced. It is important to appreciate that the Trained animals do not express fear responses or stress, and while the Yoked animals also do not express fear behavior, their corticosterone levels transiently elevate during the initial training but are at baseline during the retention test. The physical experience of the environment of the three different groups of mice is identical during the retention test and their circulating levels of corticosterone are indistinguishable at the time of post-retention euthanasia [20]. Mice were euthanized either 1 h after the third training trial (see timeline, Fig 1A) or 1 h after the 24 h retention test. In a second series of experiments, mice that received intrahippocampal injection of the Pol I inhibitor, CX-5461 were compared to control mice that received a vehicle injection (see details below).

Stereotaxic intrahippocampal cannulation surgery and drug injection

A detailed procedure for implanting guide cannulas and performing intrahippocampal injections has been published [17]. In preparation for stereotaxic surgery, anesthesia was induced by a mixture of ketamine (75mg/kg) and dexmedetomidine (1 mg/kg) delivered by intraperitoneal (IP) injection (total volume: 200 μl / 20 mg mouse). The anesthetized mice were mounted in a stereotaxic frame to implant a pair of guide cannula with the tip above the injection target: the CA1 region of the dorsal hippocampus (Bregma coordinates: AP -1.94mm, ML ± 1.00mm, DV -0.90mm). The implanted injection hardware was manufactured by Plastics One, Roanoke, VA (Part Numbers: C235GS-5-2.0, C235DCS-5, 303DC/1, C235IS-5; guide cannula, cannula dummy, cannula cap, injection needle, respectively). Antisedan (0.65 mg/kg IP) was administered to reverse the anesthesia at the end of surgery. The mice were provided with orally available post-surgical analgesia (Ketaprofen) daily for 5 days and allowed a total of 4-6

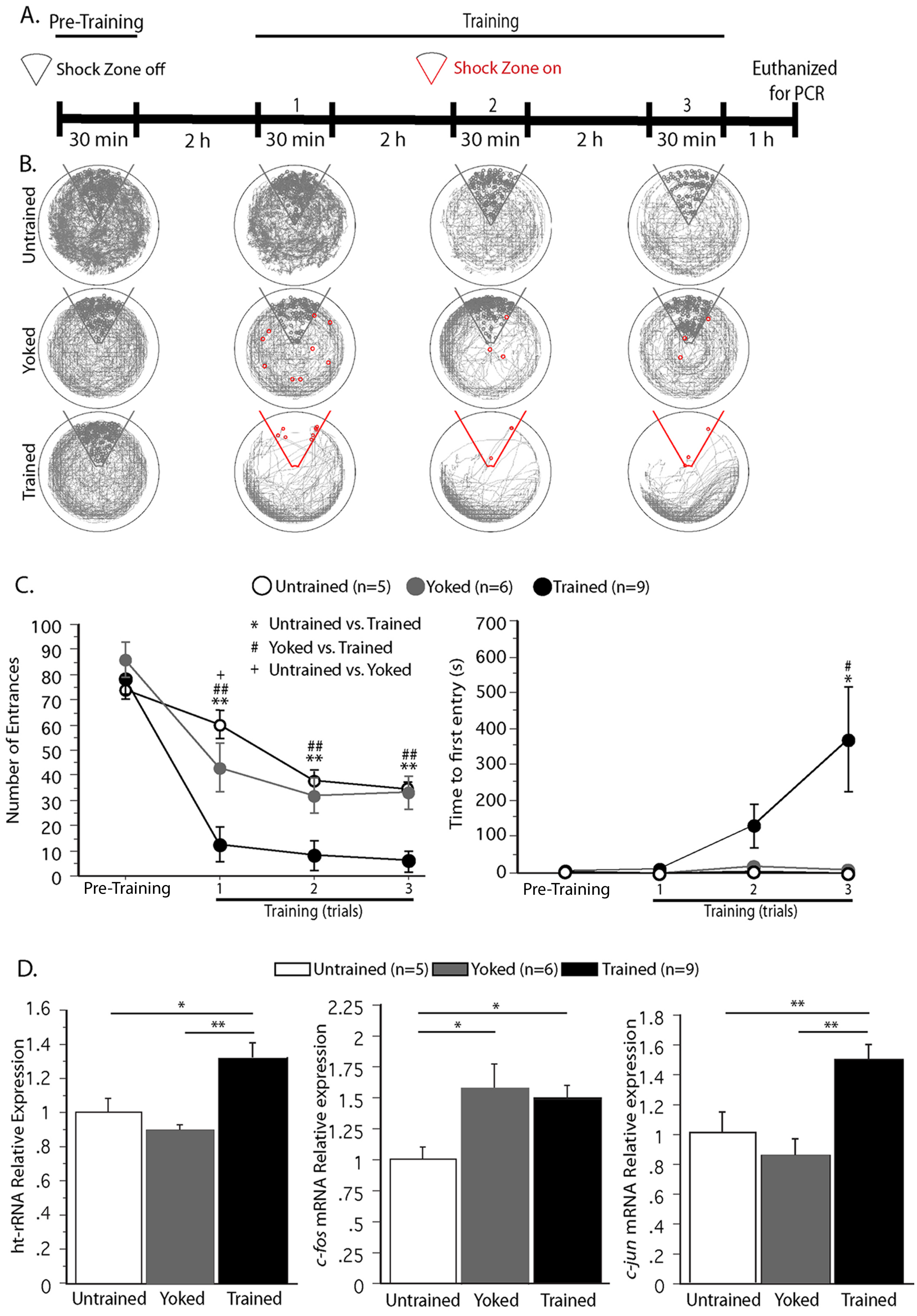
A.
Pre-Training
Training
Shock Zone off
Shock Zone on
Euthanized for PCR
1
2
3
30 min
2 h
30 min
2 h
30 min
2 h
30 min
1 h
B.
Untrained
Yoked
Trained
C.
Untrained (n=5)
Yoked (n=6)
Trained (n=9)
* Untrained vs. Trained
# Yoked vs. Trained
+ Untrained vs. Yoked
Number of Entrances
Time to first entry (s)
Pre-Training
Training (trials)
D.
Untrained (n=5)
Yoked (n=6)
Trained (n=9)
ht-rRNA Relative Expression
c-fos mRNA Relative expression
c-jun mRNA Relative expression
Untrained
Yoked
Trained

Fig 1. Ribosomal RNA (rRNA) synthesis is induced by spatial memory training. A) Timeline of training protocol. B, C) APA behavioral data from each 30 min trial (pre-training, and training trials 1, 2, and 3). Three groups of mice were analyzed: 1) Untrained, 2) Yoked, and 3) Trained. B) Representative paths (grey tracings) of individual animals during pre-training and each 30 min training trial. Red circles indicate the animal's location when a shock was received. Grey circles indicate the location of the animal when it would have received a shock had the shock zone been active. C) *Left*, The number of entrances into the shock zone during pre-training and each training trial. Only the APA trained mice (black circles) learned to actively avoid the zone. *Right*, Time to first entry into the shock zone was significantly higher for the Trained group beginning with the 2nd training trial, compared to Yoked and Untrained controls. D) Relative levels of gene expression in mouse dorsal hippocampi one hour after third training trial, as determined by Real-time qPCR. *Left*, Precursor rRNA (*ht-rRNA*) expression is upregulated only in the Trained group, not in Untrained or Yoked controls. *Middle*, The IEG, *c-fos*, known to be activated by both learning and task related stress is elevated in both Yoked and APA trained mice compared to Untrained controls. *Right*, The IEG, *c-jun*, activated by learning but not associated stress, similarly to rRNA, is up-regulated in the Trained group but not the Untrained or Yoked controls. $^{\#,+,*}$ = p<0.05, $^{\#\#,++,**}$p<0.01.

https://doi.org/10.1371/journal.pone.0203374.g001

weeks to fully recover prior to behavioral training. Intracranial injection: 30 min prior to pre-training, mice were bilaterally injected with 1 μl (0.5 μl each side) of vehicle (0.1% DMSO in sterile PBS) or the Pol I inhibitor, CX-5461 (1μM in vehicle) by inserting the injection needles into the guide cannula.

Statistics

Student's t tests (when comparing two groups) or ANOVA (when comparing 3 or more groups) were performed to compare expression levels by qPCR. For behavioral data, multi-factor comparisons were performed using ANOVA with repeated measures, as appropriate. Post-hoc multiple comparisons were performed by Tukey tests after ANOVA. Statistical significance was accepted at $p < 0.05$.

Results

New rRNA synthesis is induced by APA training

Previously, we demonstrated *in vitro* that nucleolar integrity and the synthesis of new PARP-1 dependent rRNAs are required for the maintenance of long-term synaptic plasticity [13, 15]. Here we asked, "*Does this occur in vivo*?" Specifically, "*Does behavioral training induce new rRNA synthesis in mice*?" We chose the hippocampus- dependent APA spatial learning task to address these questions. One of the advantages of the APA task is that it can be used to differentiate between multiple aspects of spatial learning and memory [17]. For instance, the average number of entrances (NE) into the shock zone during each training trial measures learning within a single session. The time to first entry (TFE) into the shock zone measures between-session (2 h) memory because it is the memory of the prior session(s) that will cause the animal to delay entry into the shock zone during the current session. We compared three behavioral groups: 1) "Untrained" animals (exploration/novelty without shocks); 2) "Trained" animals (shocked when entering the shock zone); and 3) "Yoked" animals that received shocks in random locations. As shown in Fig 1, only the Trained animals learned to avoid the shock zone; (compare the trajectories of representative Trained, Yoked, and Untrained animals in Fig 1B). The steep reduction in NE by the Trained group also reflects rapid place avoidance, as confirmed by two-way (group x trial) repeated measures ANOVA. There were significant effects of group ($F_{2,18} = 18.22$; $p < 0.001$; post-hoc Tukey's test confirmed Trained < Untrained = Yoked), trial ($F_{3,54} = 122.00$; $p < 0.001$) and the group x trial interaction ($F_{2,18} = 9.28$; $P < 0.001$; post-hoc Tukey's test confirmed Trained < Untrained = Yoked) (Fig 1C, left). Overall, the trials show no difference between Untrained and Yoked groups; however, a difference is observed between Untrained and Yoked in the first training trial (Fig 1C, left). The difference disappears when the NE is normalized to path length (data not shown) suggesting that it is a product of the behavioral response of Yoked mice to being shocked. The between-session (2 h) memory is assessed by the

TFE (Fig 1C, right). On the first training trial, none of the animals had any prior experience with shock and accordingly, there were no differences in TFE between the three groups. This begins to change in Trial 2 when the Trained animals can use the memory of Trial 1 shock locations to avoid entering the shock zone, as indicated by the non-significant rise in TFE of Trained animals compared to the Yoked and Untrained controls (Fig 1C, right). By the third training trial there TFE is significantly elevated in the Trained group compared to the two control groups that showed no change in TFE throughout the experiment (Fig 1C, right). There was no difference in TFE between Untrained mice with nothing to learn and Yoked mice with no place to avoid (Fig 1C, right). These impressions were confirmed by two-way (group x trial) repeated measures ANOVA, followed by post-hoc Tukey's test. There were significant effects of group ($F_{2,18} = 4.35$; $p = 0.03$; Untrained = Yoked < Trained); however, there were no significant effects of the trial ($F_{3,54} = 1.76$; $p < 0.17$). Likewise, there was no significant interaction between the two variables (group X trial) ($F_{2,54} = 2.09$; $p = 0.073$). These results demonstrate that the Trained group formed a 2 h place avoidance memory whereas there was no evidence of a place memory in the two control groups (Untrained and Yoked) (Fig 1C).

Does long-term APA memory formation induce new rRNA synthesis?

We next evaluated the effect of training on rRNA gene expression by collecting tissue from the same three groups of mice 1 h after the third training trial for RNA analysis (Fig 1A). To distinguish newly synthesized rRNA from pre-existing rRNA, we designed primers to detect the unedited 45S rRNA precursor (ht-rRNA) [15]. Precursor ht-rRNA was upregulated in the Trained but not the Untrained or Yoked groups (ANOVA: $F_{2,21} = 8.17$; $p < 0.01$, Trained > Yoked = Untrained) (Fig 1D, left). We also examined whether training activates the Immediate Early Genes (IEGs) *c-fos* and *c-jun*, which are markers of neuronal activation (Fig 1D). Whereas both the Trained and Yoked groups expressed increased levels of *c-fos* compared to Untrained mice, (ANOVA: $F_{2,21} = 4.145$; $p = 0.03$, Trained = Yoked > Untrained) (Fig 1D, *middle*), only the Trained group expressed increased levels of *c-jun* (ANOVA: $F_{2,21} = 9.43$; $P < 0.001$, Trained > -Yoked = Untrained) (Fig 1D, right). These results are consistent with previous studies showing that while *c-fos* expression gets activated with stress and learning, *c-jun* expression gets activated only with learning [20–22].

Variant IV activity-dependent rRNA gene expression is differentially regulated after APA memory formation

Ribosomal rRNA genes (rDNA) have multiple copies within the genome. In mice, the copies have been grouped into seven different variants (v-RNA I -VII), that were found to be epigenetically regulated in various tissues. In brain, v-RNA**s** I, II, III, IV and VI are expressed, while v-rRNAs V and VII are silent [23]. Given the increase in rRNAs observed after training, we asked: *Are all of the known variants induced by learning, or only a subset?*

Using qPCR, we compared the levels of expression of different rDNA variants in dorsal hippocampi of Trained and Untrained animals one hour after learning. Only one of the known hippocampal variants, v-rRNA IV, was significantly up-regulated after APA training compared to Untrained controls (Fig 2, $t_{11} = 3.42$; $^{*}p<0.01$), indicating that v-RNA IV expression is correlated with memory formation.

Training–induced rRNA synthesis is required for memory formation

Nucleolar activation and rRNA synthesis are early and essential steps in the production of new ribosomes [9]. Given the abundance and longevity of rRNAs and ribosomes in neurons and other tissues [24–26], we wondered: *Why does training induce de novo rRNA synthesis? Is it memory formation, or persistence that requires new rRNA synthesis*? To find out, we

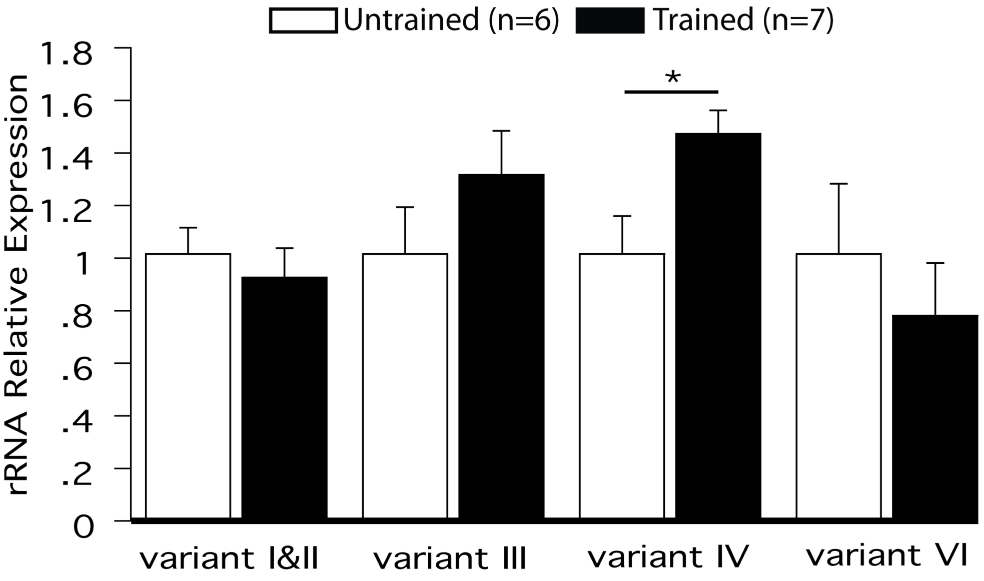

Fig 2. rRNA variant IV expression is upregulated one hour after learning. Real-Time qPCR relative expression show that of the five known hippocampal rRNA variants only variant IV is significantly up-regulated in the Trained group one hour after the third APA training trial: $T_{11} = 3.42$; $^{*}p<0.01$.

https://doi.org/10.1371/journal.pone.0203374.g002

administered either CX-5461 (n = 12) or vehicle (n = 11) by intrahippocampal injection 30 min prior to APA training (See Timeline Fig 3A). In order to evaluate gene expression, half the cohort (n = 6 for both vehicle and CX-5461 treated mice) were euthanized one hour after the last training trial while the other half (n = 6 for vehicle, n = 5 for CX-5461 treated mice) were tested for memory retention 24 h later. Learning by CX-5461-treated and vehicle-treated control mice was indistinguishable as illustrated by the representative trajectories of the animals during each training trial (Fig 3B) and the NE (Fig 3C, left; group: $F_{1,22} = 0.79$; $p > 0.38$; trial $F_{3,66} = 211.70$; $p < 0.001$; group x trial interaction $F_{3,66} = 0.43$; $p = 0.70$). Note that both groups learn to stay approximately opposite the shock zone (4 to 8 O'clock; Fig 3A, bottom). There were no group differences in between-trial memory as indicated by the TFE (Fig 3C, right; group: $F_{1,22} = .06$; $p > 0.8$; trial $F_{3,66} = 19.32$; $p < 0.01$; group x trial interaction $F_{3,66} = 0.07$; $p = 0.79$). These data suggest that Pol I activity is not required for spatial learning.

In order to test LTM, half of the original cohort (6 vehicle and 5 CX-5461 injected mice) was returned to their cages after training and given a retention test 24 h later. The retention test is performed by returning the animal to the rotating arena for 10 min with the shock zone turned off. If the animal remembers the previous training, it will avoid the inactive shock zone. The movement of the animal is tracked and recorded by an overhead camera and TrackAnalysis (Bio-Signal Group) software (See Materials and methods). LTM is indicated by the trajectory of the animal (Fig 3B bottom right, grey tracings) and quantified by the time to first entry (TFE) into the deactivated shock zone (Fig 3C, right). One-day memory was impaired in the Pol I inhibited group compared to the vehicle control. As shown in Fig 3A (bottom right), during the 10 min retention test, the animals receiving vehicle largely avoid the location where shocks were previously experienced, whereas the animals treated with the Pol I inhibitor do not; instead, their behavior resembles that of animals during pre-training, before any shock was experienced. This is not merely rapid extinction of the active place avoidance memory because the TFE in the Pol I inhibited group was significantly lower than it was for the vehicle-injected group (Fig 3C, far right; $F_{1,9} = 3.57$; $p < 0.05$), although the TFE estimate of between-trial 2 h memory did not differ between the groups (Fig 3C, left). In fact, TFE in the Pol I

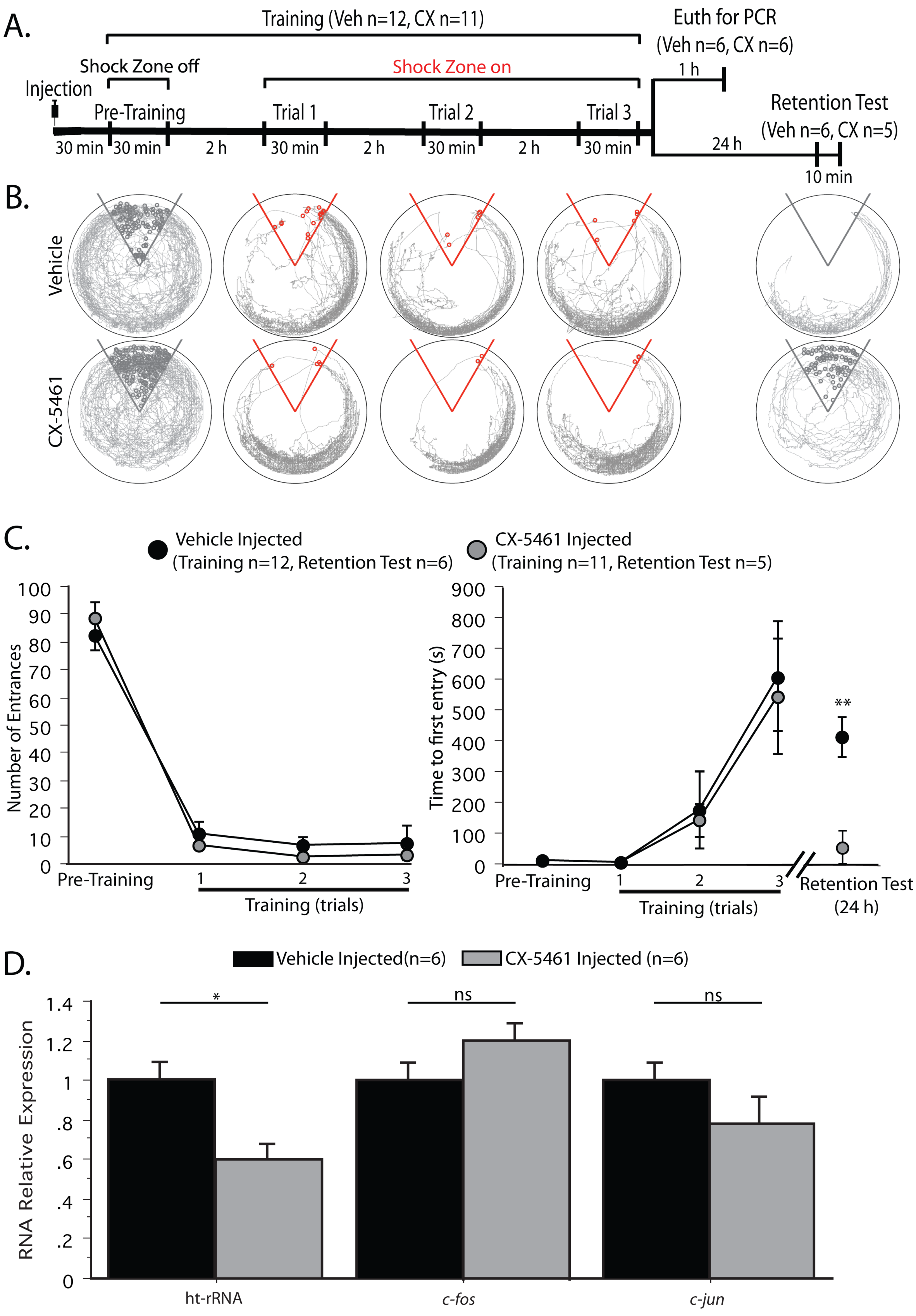
A.
Training (Veh n=12, CX n=11)
Euth for PCR
(Veh n=6, CX n=6)
Shock Zone off
Shock Zone on
Injection
1 h
Pre-Training
Trial 1
Trial 2
Trial 3
Retention Test
(Veh n=6, CX n=5)
30 min
30 min
2 h
30 min
2 h
30 min
2 h
30 min
24 h
10 min
B.
Vehicle
CX-5461
C.
Vehicle Injected
(Training n=12, Retention Test n=6)
CX-5461 Injected
(Training n=11, Retention Test n=5)
Number of Entrances
Pre-Training
Training (trials)
Time to first entry (s)
Retention Test
(24 h)
**
D.
Vehicle Injected(n=6)
CX-5461 Injected (n=6)
RNA Relative Expression
*
ns
ns
ht-rRNA
c-fos
c-jun

Fig 3. RNA Pol I inhibitor, CX-5461, disrupts consolidation of long-term memory, but not learning. A) Timeline of the training protocol. Note that half of the animals were euthanized 1 h after the third training trial for qPCR analysis (see 3D) while the remainder were given a retention test 24 h later (see 3C). B) Representative paths (trajectories) of animals during pre-training, training and the 10 min retention test given 24 h after the 3rd training trial (grey tracings). Red circles indicate the animal's location when a shock was received. Grey circles indicate the position of the animal where it would have received a shock had the shock zone been active. C) Comparison of learning and memory between APA trained mice who received intracranial injection of vehicle (black circles) and CX-5461 (grey circles). *Left*, Number of entrances during the 30 min trials. No significant difference in the number of entrances indicates equivalent learning of CX-5461 treated animals and vehicle-treated controls. *Right*, Time to first entry the shock zone during each training trial and on the 24 h retention test. There were no significant differences in between-trial memory or between the two groups during training. In contrast, the vehicle injected animals continue to show place avoidance memory 24 h later; whereas CX-5461 injected animals do not. D) Real-Time qPCR relative expression shows that CX-5461 has no effect on Pol II dependent *c-fos* and *c-jun* transcription, but does inhibit Pol I dependent rRNA transcription. $^{*}p<0.05$, $^{**}p<0.01$.

https://doi.org/10.1371/journal.pone.0203374.g003

inhibitor group was indistinguishable from pre-training–before any shock was experienced (paired t test: t_4 = -1.06, p = 0.35), indicating that inhibition of rRNA synthesis results in a failure of 24 h memory expression.

CX-5461 is a direct inhibitor of the Pol I machinery and acts by preventing the formation of the Pol I preinitiation complex while leaving Pol II transcription, DNA replication, and translation processes intact [27]. In a previous study, we tested CX-5461 *in vitro* and found that the same treatment that effectively suppressed Pol I-directed rRNA synthesis had no effect on Pol II dependent, plasticity-induced IEG expression [15]. To investigate the selectivity of the CX-5461 treatment used in this study, the training–induced expression of Pol I dependent ht-rRNA and Pol II dependent *c-fos* and *c-jun* mRNAs in dorsal hippocampus was assessed by qPCR in the subset of mice that were euthanized 1 h after the third training trial (Fig 3D). As expected, CX-5461 had no effect on the activity-dependent expression of *c-fos* mRNA (t_{10} = 1.31; p = 0.22) or *c-jun* mRNA (t_{10} = 1.32; p = 0.22). In contrast, the training-induced increase in total precursor rRNA observed in the vehicle-treated group was effectively suppressed by the pre-training CX-5461 injection (Fig 3D; t_{10} = 2.76; $p < 0.05$). In order to determine whether the failure of CX-5461 treated mice to consolidate memory was due to irreversible toxic changes induced by the drug, we subjected mice to intrahippocampal injection of vehicle or CX-5461 and then waited 48 h before APA training. Both groups learned the task efficiently and were able to consolidate the memory as indicated by the retention test given 24 h later (See S1 Fig). These results demonstrate that CX-5461 does not permanently impact the animal's ability to learn or remember. The fact that 24 h memory is impaired by blocking new, activity-dependent rRNA synthesis during learning, suggests that pre-existing ribosomes cannot substitute for activity-dependent, new ribosomes when it comes to memory consolidation and perhaps maintenance. It is interesting to note that the window for the necessary Pol I directed gene expression appears to be within hours of the training experience as opposed to days.

Inhibition of activity-dependent expression of v-rRNAs disrupts memory

Next, we examined the pattern of v-rRNA expression in the same cohort of vehicle and CX-5461 injected mice that provided data for Fig 3D. Again, the gene expression level of the vehicle-injected control group was set to one for comparison with the CX-5461 injected group (Fig 4). Like their non-surgery Trained counterparts (refer to Figs 1 and 2), the vehicle-injected group showed a learning-induced upregulation of v-rRNA IV while the other tested variants remained unchanged (Fig 4, black bars).

The persistence of learning induced nucleolar activation is long lasting

To distinguish between maintained gene expression and an effect of recall, we evaluated gene expression in four groups of mice, Trained and Untrained mice, with and without having been given a memory retention test 24 h after the third training trial (Fig 5A top). Fig 5B shows the

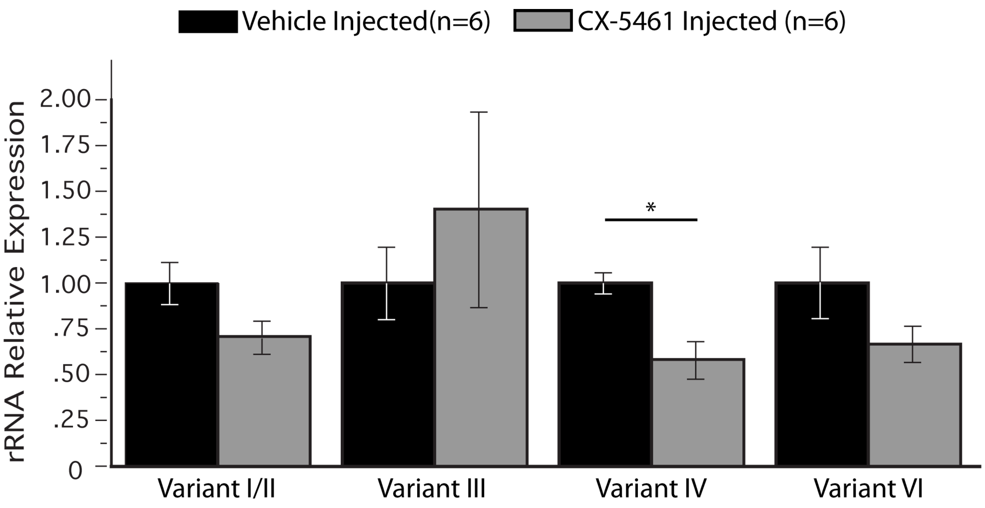

Fig 4. Pol I inhibition before and during training prevents learning-induced upregulation of rRNA Variant IV. Real-Time qPCR samples derived from dorsal hippocampi collected 1 h after APA training taken show that CX-5461 only significantly inhibits the rRNA variant IV after training. $t_{10} = 3.96$; $^{*}p<0.01$.

https://doi.org/10.1371/journal.pone.0203374.g004

relative expression of 45S rRNA precursor in each group. There was no significant difference between mice that did or did not receive a retention test (n = 5, each group). Nevertheless, rRNA precursor more than doubled in both Trained groups compared to the Untrained control groups (n's = 5, two-way group x treatment ANOVA: group: $F_{1,16} = 26.21$; $p < 0.001$; treatment: $F_{1,16} = 0.70$; p = 0.41; interaction: $F_{1,16} = 0.07$; p = 0.79). These results indicate that the learning-induced upregulation of rRNA synthesis observed 24 h after training does not seem to be an effect of memory recall or the experience of the retention test, but instead is a learning-induced upregulation of nucleolar activity that is maintained at least 24 h after memory training. Interestingly, the sustained increase in precursor rRNA cannot be explained by an upregulation of any of the known rRNA variants (S2 Fig), suggesting that yet unidentified variants may play a role in memory maintenance. Another way to assess nucleolar activity is by immunohistochemistry (IHC). Increases in nucleolar activity (rRNA synthesis and ribosomal biogenesis) is accompanied by observable changes in structure and composition that manifest as an increase in size and the recruitment of nucleolar proteins. Fibrillarin is a nucleolus specific protein that plays a key role in processing nascent precursor rRNAs making it a sensitive marker of nucleolar activity. Fig 5C shows IHC of dorsal CA1 from APA trained and untrained mice that were euthanized 24 h after training, with or without having received a retention test. The data was analyzed using a two-way ANOVA in which training and retention test were the two parameters (see S1 File). We found that the average intensity of nucleolar fibrillarin is significantly increased 24 h after APA exposure in trained animals compared to untrained controls ($F_{1,8}$ = 28.484, p = .001). We also found a smaller, but significant effect of the retention test, independent of training ($F_{1,8} = 7.351$, p = .027; Fig 5C and S3 Fig). We did not find a difference in the interaction of training with retention test ($F_{1,8} = 3.405$, p = .102). Together with the qPCR results, these data indicate that the upregulation of nucleolar activity and rRNA synthesis that is observed an hour after training is long-lasting, persisting at least one day.

Discussion

The role of the nucleolus and Pol I transcription in synaptic plasticity and learning and memory, has scarcely been explored. Recently, it was shown that induction of long-term facilitation in invertebrates [13] and long-term potentiation (LTP) in vertebrates [15], produces a PARP-

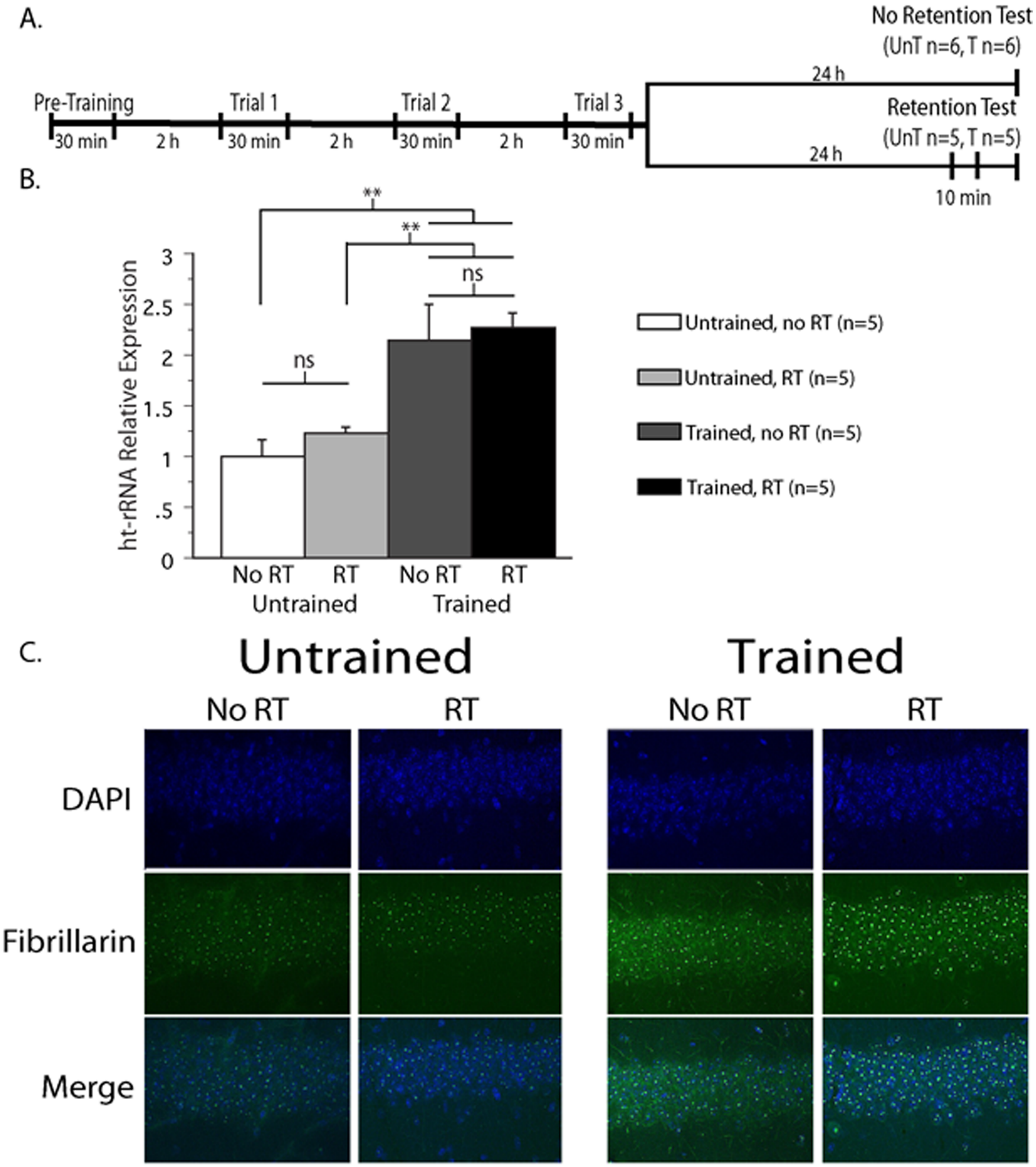

Fig 5. Elevated expression of rRNA is maintained at least 24 h after training. A) Timeline of the training protocol. Note that half of the animals were given a retention test, while the other half were not. All animals were euthanized 24 h after training. B) Expression of ht-rRNA precursor 24 h after APA exposure. 45S ht-rRNA expression is elevated 24 h after training compared to Untrained animals. The retention test did not significantly affect rRNA expression. C) Immunohistochemistry of dorsal CA1 fixed 24 h after training with or without a retention test. Increased fibrillarin staining in the Trained animals indicates an active nucleolus. $^{**}p<0.01$.

https://doi.org/10.1371/journal.pone.0203374.g005

1-dependent activation of rRNA synthesis in the neuronal nucleolus. This activation has been shown to be necessary for late-phase LTP, an *ex vivo* model of memory [15]. These results led to the prediction that nucleolar integrity and activity-dependent rRNA synthesis would also be required for LTM, *in vivo*. We explored this possibility using the hippocampal dependent APA

spatial memory task and intra-hippocampal injection of the specific Pol I inhibitor, CX-5461, and found that—indeed, activity-dependent rRNA synthesis is required for 24 h memory, and that training-induced rRNA synthesis is differentially regulated and long-lasting (24 h). In a recent study of learning-induced gene expression in mouse cortical and subcortical networks, Capitano and colleagues [28] used a massed version of the Morris Water Maze (MWM) task and post-training intracranial injection of CX-5461 to look at the role of rDNA expression in spatial memory formation. In spite of the differences in methodology, the study produced two complimentary findings: 1) spatial memory training induces a transient increase in de novo rRNA synthesis in the ventral hippocampus; and 2) inhibition of rRNA synthesis in the dorsal hippocampus prevents memory consolidation [28]. Further analysis will be necessary to determine which forms of long-term synaptic plasticity and memory require nucleolar activity and rRNA expression, and to what degree (e.g. transient or long-lasting) is de novo rRNA expression required for different forms of memory.

Quantitative versus qualitative

I. Are all ribosomes created equal?. Plasticity-dependent rRNA synthesis, ribosome biogenesis is required for LTP [15] and for LTM (Fig 3). Is this because the quantity of ribosomes under basal conditions is limiting, or are stimulation-induced ribosomes qualitatively different? Consider the case for quantity: neurons may simply need more ribosomes to meet the translation demands of long-term plasticity and memory. Changes in the translational capacity of cells in response to nutrient availability or growth phase transitions, have been tracked by shifts in ribosome optical density profiles that detect inactive 40S and 60S ribosome subunits, and active 80S monosomes and polysomes [29]. Under normal physiological conditions there is typically a fraction of ribosomes that are not actively engaged in translation. This reserve of inactive ribosomes ('the 40S and 60S pool') allows cells to rapidly accommodate increased demand for protein synthesis. Both rRNAs and ribosomes are quite stable with an average ribosomal half-life of 5-10 days [25, 26, 29, 30]; and there is indirect evidence that they can persist for months [24, 31]. Previously, we found in mouse hippocampal slices that if Pol I activity is blocked, LTP can be initiated but not maintained [15]. Here we find that if Pol I activity is blocked during training, the animals still learn but will not exhibit memory 24 h later.

If all ribosomes are equal and interchangeable, then why can't pre-existing ribosomes mitigate the effect of acute Pol I inhibition? *Why can't they at least partially compensate when synthesis is inhibited*? One possibility is that at the molecular level, long-term plasticity and memory are *all-or-nothing* events and that the ribosomal reserve is insufficient to reach the required threshold of translational capacity–hence the need for *de novo* rRNA synthesis and ribosome biogenesis. Alternatively, new learning-induced ribosomes might be required because they are *qualitatively* different. There are several potential sources of ribosomal heterogeneity ranging from ribosomal protein composition [32] to differentially expressed rDNA sequence variants as has been described in humans [33–36]. It is possible that learning-induced v-rRNA IV is one such sequence variant. Over all, the potential for functional sequence variation among differentially regulated rDNA transcription units remains unexplored. Nevertheless, the data presented here supports a model in which a subset of learning-induced rRNAs, expressed within an hour after training, are required to form a memory that can persist days. Further studies are needed to determine whether the functional differences between learning-induced ribosomes (required for memory) and pre-existing ones (unable to compensate), stem from a qualitative change in the rRNAs and/or protein composition, or simply the temporal context of a quantitative change in ribosome number.

II. Functional rRNA expression variants. While there is biological precedence for rRNA sequence variants (reviewed in [37]), it is also possible that the specific sequence of the rRNA transcript that is produced is not as important as the temporal and physiological context of its expression. For example, in the protozoan, *Plasmodium berghei*, rRNA gene expression switches between different rDNA transcription units in a developmental-stage and species-specific manner [38]. The rRNAs expressed are structurally distinct, suggesting that the ribosomal subunits they form may also be structurally and thus, functionally distinct. Differentially expressed rRNA variants (v-rRNAs) have been documented in more complex organisms ranging from *Arabidopsis* [39] to zebrafish [40], to mice [23, 41, 42] to humans [33, 35, 36, 43]. Functional sequence variants of 5S rRNA have been described for the sea urchin, *P. lividus* l [44]. More recently, the same laboratory characterized three clusters of 18S-26S rRNA genes with different Non-Transcribed Spacer (NTS) regions that are important for the epigenetic regulation and coordination of large and small sub-unit rRNA synthesis required for making new ribosomes [45]. In mice, seven epigenetically regulated v-rRNAs have been cloned and shown to be either constitutively silent or differentially expressed in various tissues [23]. In this study, we found that one of the v-rRNAs described by Tseng and colleagues [23], v-rRNA IV, is specifically upregulated in the dorsal hippocampus by learning, suggesting that different variants may be differentially regulated in response to different types of neuronal stimuli, and may have different transcriptional or epigenetic elements regulating their expression. Previously we found that plasticity-induced rRNA synthesis requires the activity of the chromatin remodeling enzyme, PARP-1 [13, 15]. These data are especially important in combination with the observation that v-rRNA IV was the only known variant that was upregulated 1 h after training, and therefore the only variant that can account for the coincident increase in ht-rRNA. These findings render v-rRNA IV a key candidate for a plasticity-induced, PARP-1 dependent rRNA variant after learning. On the other hand, the discrepancy observed between the ht-rRNA and the known v-rRNAs (compare Fig 5B and S2 Fig) expression suggests that an unknown uncharacterize v-rRNA accounts for the 24 h increase expression of ht-rRNA. It is important to notice that our v-rRNAs quantification it is based upon a partial characterization of vDNAs in mouse [23]. Recently, Mathew Parks and colleagues used whole-genome sequencing data and rRNA expression analysis to identify variants expressed in different tissue types [33]. Further analysis and characterization of vDNAs and their expression is necessary to assess plasticity dependent v-rRNAs. Another interesting observation is the effect of recall on fibrillarin staining (S3 Fig). Although smaller than the effect of training, a significant effect was found, suggesting that biogenesis of ribosomes may also occur in response to recall.

The nucleolus and cognition—Implications for Alzheimer's disease

Over the last decade, there has been a growing recognition of the importance of the nucleolus as an indicator of neuronal health and disease [46–48]. Studies of neurodegenerative disorders such as Alzheimer's, Huntington's and Parkinson's diseases reveal nucleolar dysfunction and aberrant rDNA methylation as common features ([49, 50, 51] respectively). In the case of Alzheimer's disease (AD), both occur in the earliest stages of pathology, making them "early markers" of AD [46, 49]. Previously, we showed *in vitro* that plasticity-dependent rRNA synthesis requires the epigenetic regulator, PARP-1 [13, 15]. Recently, in a histological study of AD that focused on nucleolar activity, we found that nucleolar localization of PARP-1 is significantly decreased in hippocampal pyramidal cells in autopsy samples from AD patients compared to age-matched controls [52]. We proposed then that PARP-1 displacement from the nucleolus leads to hypermethylation of rDNA resulting in the downregulation of rRNA synthesis and ribosome biogenesis. Without new ribosomes, the synthesis of new proteins

becomes impaired and the formation of new memories disrupted. Central to this hypothesis is the assertion that the nucleolus plays a key role in cognition. Rather than just being a marker of disease, we propose that loss of nucleolar activity is an essential part of the cognitive deficits observed in AD [37, 52]. In support of this view, a recent article by Li and collaborators identified a nucleolar-specific long non-coding RNA (LoNA) regulating rRNA synthesis during synaptic plasticity and learning and memory [53]. Downregulation of LoNA enhances long-term memory in WT mice, as well as restores impaired memory function in APP/PS1 transgenic mice. Whether or not PARP-1 downregulates LoNA to regulate rRNAs expression during synaptic plasticity and learning and memory has not been determined yet. Taken together, these data strongly support an essential role for nucleolar integrity and experience-induced rRNA synthesis in memory and cognition. This model predicts that, regardless of the source of nucleolar stress and dysfunction, therapeutic approaches aimed at restoring nucleolar activity will be necessary to restore cognition.

Supporting information

S1 Fig. Intra-hippocampal treatment of CX-5461 48 h before training does not inhibit 24 h memory consolidation. A) Timeline of the training protocol. Animals were injected with CX-5461 or vehicle 48 h before commencement of training. B) Comparison of learning and memory between APA trained mice who received intracranial injection of vehicle (black circles) and CX-5461 (grey circles). *Left*, Number of entrances during the 30 min training trials. *Right*, Time to first entry into the shock zone during each training trial and the 24 h retention test. Both CX-5461 and vehicle injected animals significantly reduced the number of entrances during training, and increased their time to first entrance indicating that they learned the location of the shock zone. A significant increase in the time to first entry during the retention test indicates that both groups exhibited memory of the shock zone 24 h after training. No significant differences in the memory retention test were observed between the two groups indicating that the group treated with CX-5461 does not lose the ability to consolidate memory.
(TIF)

S2 Fig. Characterized rRNA variants are not differentially expressed 24 h after training. Real-Time qPCR analysis demonstrates that none of the five known hippocampal rRNA variants are significantly upregulated 24 h after the third APA training trial, whether or not an animal received a retention test. [Significance analyzed by ANOVA. All p values > 0.14].
(TIF)

S3 Fig. Hippocampal neuronal nucleolar expression of fibrillarin is increased after training and retention test. A) Table showing mean (and standard deviation) for each of 4 groups (n = 3/group) (trained and untrained with and without RT), as well as total for each condition. A two-way ANOVA found increased fibrillarin staining in response to training ($F_{1,8} = 28.484$, $p = .001$) and RT ($F_{1,8} = 7.351$, $p = .027$), but not the interaction of the two Training*RT ($F_{1,8} = 3.405$, $p = .102$). B) Graphical representation of the same data showing an effect of RT (right panel compared to left) and training (black dots compared to grey). Each dot represents the average fibrillarin intensity of an animal.
(TIF)

S1 File. Supporting information methods. Description of the methods used to produce the data presented in S1–S3 Figs.
(DOCX)

S2 File. Primary data. The primary data points used to produce means and variance presented in figures and results.
(XLSX)

Acknowledgments

We would like to thank Drs. Jenny Libien, Juan Marcos Alarcon and Samuel Schacher for reading the manuscript critically.

Author Contributions

Conceptualization: Kim D. Allen, Matthew J. Regier, Jason Wolk, A. Iván Hernández.

Data curation: Kim D. Allen, Matthew J. Regier, Changchi Hsieh, Panayiotis Tsokas, Maya Barnard, Shwetha Phatarpekar, André A. Fenton, A. Iván Hernández.

Formal analysis: Kim D. Allen, Matthew J. Regier, Panayiotis Tsokas, Todd C. Sacktor, André A. Fenton, A. Iván Hernández.

Investigation: Kim D. Allen, Matthew J. Regier, Maya Barnard, Shwetha Phatarpekar, Jason Wolk, A. Iván Hernández.

Methodology: Kim D. Allen, Matthew J. Regier, Changchi Hsieh, Panayiotis Tsokas, Maya Barnard, Jason Wolk, Todd C. Sacktor, André A. Fenton, A. Iván Hernández.

Project administration: A. Iván Hernández.

Resources: Todd C. Sacktor, André A. Fenton, A. Iván Hernández.

Supervision: Kim D. Allen, A. Iván Hernández.

Validation: Kim D. Allen, Maya Barnard, A. Iván Hernández.

Visualization: Kim D. Allen, A. Iván Hernández.

Writing – original draft: Kim D. Allen, A. Iván Hernández.

Writing – review & editing: Matthew J. Regier, Changchi Hsieh, Panayiotis Tsokas, Todd C. Sacktor, André A. Fenton, A. Iván Hernández.

References

1. Katz JJ, Halstead WC. Protein organization and mental function. Comp Psychol Monographs. 1950; 20: 1–38.
2. Flexner JB, Flexner LB, Stellar E. Memory in Mice is Affected by Intracerebral Puromycin. Science. 1963; 141:57–59. PMID: 13945541
3. Castellucci VF, Frost WN, Goelet P, Montarolo PG, Schacher S, Morgan JA, et al. Cell and molecular analysis of long-term sensitization in Aplysia. Journal de physiologie. 1986; 81:349–357. PMID: 3572827
4. Schacher S, Castellucci VF, Kandel ER. cAMP evokes long-term facilitation in Aplysia sensory neurons that requires new protein synthesis. Science. 1988; 240:1667–1669. PMID: 2454509
5. Kang H, Schuman EM. A Requirement for Local Protein Synthesis in Neurotrophin-Induced Hippocampal Synaptic Plasticity. Science. 1996; 273:1402–1406. PMID: 8703078
6. Alberini CM. Transcription Factors in Long-Term Memory and Synaptic Plasticity. Physiological Reviews. 2009; 89:121–145. https://doi.org/10.1152/physrev.00017.2008 PMID: 19126756
7. West AE, Greenberg ME. Neuronal Activity–Regulated Gene Transcription in Synapse Development and Cognitive Function. Cold Spring Harbor Perspectives in Biology. 2011; 3:a005744. https://doi.org/10.1101/cshperspect.a005744 PMID: 21555405

8. Grummt I. Life on a planet of its own: regulation of RNA polymerase I transcription in the nucleolus. Genes & Development. 2003; 17:1691–1702.
9. Russell J, Zomerdijk JCBM. RNA-polymerase-I-directed rDNA transcription, life and works. Trends in Biochemical Sciences. 2005; 30:87–96. https://doi.org/10.1016/j.tibs.2004.12.008 PMID: 15691654
10. Junera HR, Masson C, Geraud G, Hernandez-Verdun D. The three-dimensional organization of ribosomal genes and the architecture of the nucleoli vary with G1, S and G2 phases. Journal Cell Science. 1995; 108:3427–3441.
11. Mélèse T, Xue Z. The nucleolus: an organelle formed by the act of building a ribosome. Current Opinion in Cell Biology 1995; 7:319–324. PMID: 7662360
12. Hernández-Verdun D, Roussel P, Thiry M, Sirri V, Lafontaine DLJ. The nucleolus: structure/function relationship in RNA metabolism. Wiley Interdisciplinary Reviews RNA. 2010; 1:415–431. https://doi.org/10.1002/wrna.39 PMID: 21956940
13. Hernández AI, Wolk J, Hu J-Y, Liu J, Kurosu T, Schwartz JH, et al. Poly-(ADP-ribose) polymerase-1 is necessary for long-term facilitation in Aplysia. Journal of Neuroscience. 2009; 29:9553–9562. https://doi.org/10.1523/JNEUROSCI.1512-09.2009 PMID: 19641118
14. Goosens KA, Maren S. Long-term potentiation as a substrate for memory: Evidence from studies of amygdaloid plasticity and Pavlovian fear conditioning. Hippocampus. 2002; 12:592–599. https://doi.org/10.1002/hipo.10099 PMID: 12440575
15. Allen KD, Gourov AV, Harte C, Gao P, Lee C, Sylvain D, et al. Nucleolar integrity is required for the maintenance of long-term synaptic plasticity. PLoS ONE. 2014; 9:e104364. https://doi.org/10.1371/journal.pone.0104364 PMID: 25089620
16. Pastalkova E, Serrano PA, Pinkhasova D, Wallace E, Fenton AA, Sacktor TC. Storage of spatial information by the maintenance mechanism of LTP. Science. 2006; 313:1141–1144. https://doi.org/10.1126/science.1128657 PMID: 16931766
17. Tsokas P, Hsieh C, Yao Y, Lesburgueres E, Wallace EJC, Tcherepanov A, et al. Compensation for PKMζ in long-term potentiation and spatial long-term memory in mutant mice. eLife. 2016; 5:e14846. https://doi.org/10.7554/eLife.14846 PMID: 27187150
18. Barber RD, Harmer DW, Coleman RA, Clark BJ. GAPDH as a housekeeping gene: analysis of GAPDH mRNA expression in a panel of 72 human tissues. Physiological Genomics. 2005; 21:389–395. https://doi.org/10.1152/physiolgenomics.00025.2005 PMID: 15769908
19. Pavlowsky A, Wallace E, Fenton AA, Alarcon JM. Persistent modifications of hippocampal synaptic function during remote spatial memory. Neurobiology of Learning and Memory. 2017; 138:182–197. https://doi.org/10.1016/j.nlm.2016.08.015 PMID: 27568918
20. Lesburgueres E, Sparks FT, O'Reilly KC, Fenton AA. Active place avoidance is no more stressful than unreinforced exploration of a familiar environment. Hippocampus. 2016; 26:1481–1485. https://doi.org/10.1002/hipo.22666 PMID: 27701792
21. Cullinan WE, Herman JP, Battaglia DF, Akil H, Watson SJ. Pattern and time course of immediate early gene expression in rat brain following acute stress. Neuroscience. 1995; 64:477–505. PMID: 7700534
22. Guzowski JF, Setlow B, Wagner EK, McGaugh JL. Experience-dependent gene expression in the rat hippocampus after spatial learning: a comparison of the immediate-early genes Arc, c-fos, and zif268. Journal of Neuroscience. 2001; 21:5089–5098. PMID: 11438584
23. Tseng H, Chou W, Wang J, Zhang X, Zhang S, Schultz RM. Mouse Ribosomal RNA Genes Contain Multiple Differentially Regulated Variants. PLoS ONE. 2008; 3:e1843. https://doi.org/10.1371/journal.pone.0001843 PMID: 18365001
24. Kiryk A, Sowodniok K, Kreiner G, Rodriguez-Parkitna J, Sönmez A, Górkiewicz T, et al. Impaired rRNA synthesis triggers homeostatic responses in hippocampal neurons. Frontiers in Cellular Neuroscience. 2013; 7:207. https://doi.org/10.3389/fncel.2013.00207 PMID: 24273493
25. Nikolov EN, Dabeva MD, Nikolov TK. Turnover of ribosomes in regenerating rat liver. The International Journal of Biochemistry. 1983; 15:1255–1260. PMID: 6628827
26. Hirsch CA, Hiatt HH. Turnover of liver ribosomes in fed and in fasted rats. Journal of Biological Chemistry. 1966; 241:5936–5940. PMID: 5954370
27. Drygin D, Lin A, Bliesath J, Ho CB, O'Brien SE, Proffitt C, et al. Targeting RNA polymerase I with an oral small molecule CX-5461 inhibits ribosomal RNA synthesis and solid tumor growth. Cancer Research. 2011; 71:1418–1430. https://doi.org/10.1158/0008-5472.CAN-10-1728 PMID: 21159662
28. Capitano F, Gargiuli C, Angerilli A, Maccaroni K, Pelliccia F, Mele A, et al. RNA polymerase I transcription is modulated by spatial learning in different brain regions. Journal of Neurochemistry. 2016; 136:706–716. https://doi.org/10.1111/jnc.13504 PMID: 26708837

29. Mathis AD, Naylor BC, Carson RH, Evans E, Harwell J, Knecht J, et al. Mechanisms of In Vivo Ribosome Maintenance Change in Response to Nutrient Signals. Molecular & Cellular Proteomics. 2017; 16:243–254.

30. Warner JR. The economics of ribosome biosynthesis in yeast. Trends in Biochemical Sciences. 1999; 24:437–440. PMID: 10542411

31. Parlato R, Kreiner G, Erdmann G, Rieker C, Stotz S, Savenkova E, et al. Activation of an endogenous suicide response after perturbation of rRNA synthesis leads to neurodegeneration in mice. Journal of Neuroscience. 2008; 28:12759–12764. https://doi.org/10.1523/JNEUROSCI.2439-08.2008 PMID: 19036968

32. Xue S, Barna M. Specialized ribosomes: a new frontier in gene regulation and organismal biology. Nature Reviews Molecular Cell Biology. 2012; 13:355–369. https://doi.org/10.1038/nrm3359 PMID: 22617470

33. Parks MM, Kurylo CM, Dass RA, Bojmar L, Lyden D, Vincent CT, et al. Variant ribosomal RNA alleles are conserved and exhibit tissue-specific expression. Sci. Adv. 2018; 4:eaao0665. https://doi.org/10.1126/sciadv.aao0665 PMID: 29503865

34. Gonzalez IL, Gorski JL, Campen TJ, Dorney DJ, Erickson JM, Sylvester JE, et al. Variation among human 28S ribosomal RNA genes. Proceedings of the National Academy of Sciences. 1985; 82:7666–7670.

35. Kuo BA, Gonzalez IL, Gillespie DA, Sylvester JE. Human ribosomal RNA variants from a single individual and their expression in different tissues. Nucleic Acids Research. 1996; 24:4817–4824. PMID: 8972871

36. Gonzalez IL, Sylvester JE. Human rDNA: evolutionary patterns within the genes and tandem arrays derived from multiple chromosomes. Genomics. 2001; 73:255–263. https://doi.org/10.1006/geno.2001.6540 PMID: 11350117

37. Hernández AI, Alarcon JM, Allen KD. New Ribosomes for new memories?. Communicative & Integrative Biology. 2015; 8:e1017163.

38. Waters AP, van Spaendonk RM, Ramesar J, Vervenne RA, Dirks RW, Thompson J, et al. Species-specific regulation and switching of transcription between stage-specific ribosomal RNA genes in Plasmodium berghei. Journal of Biological Chemistry. 1997; 272:3583–3589. PMID: 9013609

39. Pontvianne F, Abou-Ellail M, Douet J, Comella P, Matia I, Chandrasekhara C, et al. Nucleolin is required for DNA methylation state and the expression of rRNA gene variants in Arabidopsis thaliana. PLoS Genetics. 2010; 6:e1001225. https://doi.org/10.1371/journal.pgen.1001225 PMID: 21124873

40. Higa-Nakamine S, Suzuki T, Uechi T, Chakraborty A, Nakajima Y, Nakamura M, et al. Loss of ribosomal RNA modification causes developmental defects in zebrafish. Nucleic Acids Research. 2012; 40:391–398. https://doi.org/10.1093/nar/gkr700 PMID: 21908402

41. Ihara M, Tseng H, Schultz RM. Expression of variant ribosomal RNA genes in mouse oocytes and preimplantation embryos. Biology of Reproduction. 2011; 84:944–946. https://doi.org/10.1095/biolreprod.110.089680 PMID: 21209414

42. Shiao Y-H, Leighty RM, Wang C, Ge X, Crawford EB, Spurrier JM, et al. Ontogeny-driven rDNA rearrangement, methylation, and transcription, and paternal influence. PLoS ONE. 2011; 6:e22266. https://doi.org/10.1371/journal.pone.0022266 PMID: 21765958

43. Gonzalez IL, Sylvester JE. Complete sequence of the 43-kb human ribosomal DNA repeat: analysis of the intergenic spacer. Genomics. 1995; 27:320–328. https://doi.org/10.1006/geno.1995.1049 PMID: 7557999

44. Dimarco E, Cascone E, Bellavia D, Caradonna F. Functional variants of 5S rRNA in the ribosomes of common sea urchin Paracentrotus lividus. Gene. 2012; 508:21–25. https://doi.org/10.1016/j.gene.2012.07.067 PMID: 22967708

45. Bellavia D, Dimarco E, Caradonna F. Characterization of three different clusters of 18S–26S ribosomal DNA genes in the sea urchin P. lividus: Genetic and epigenetic regulation synchronous to 5S rDNA. Gene. 2016; 580:118–124. https://doi.org/10.1016/j.gene.2016.01.010 PMID: 26789074

46. Parlato R, Kreiner G. Nucleolar activity in neurodegenerative diseases: a missing piece of the puzzle? Journal of Molecular Medicine. 2013; 91:541–547. https://doi.org/10.1007/s00109-012-0981-1 PMID: 23179684

47. Grummt I. The nucleolus—guardian of cellular homeostasis and genome integrity. Chromosoma. 2013; 122:487–497. https://doi.org/10.1007/s00412-013-0430-0 PMID: 24022641

48. Hetman M, Pietrzak M. Emerging roles of the neuronal nucleolus. Trends in Neurosciences. 2012; 35:305–314. https://doi.org/10.1016/j.tins.2012.01.002 PMID: 22305768

49. Pietrzak M, Rempala G, Nelson PT, Zheng J-J, Hetman M. Epigenetic silencing of nucleolar rRNA genes in Alzheimer's disease. PLoS ONE. 2011; 6:e22585. https://doi.org/10.1371/journal.pone.0022585 PMID: 21799908

50. Lee J, Hwang YJ, Ryu H, Kowall NW, Ryu H. Nucleolar dysfunction in Huntington's disease. Biochimica et biophysica acta. 2014; 1842:785–790. https://doi.org/10.1016/j.bbadis.2013.09.017 PMID: 24184605

51. Parlato R, Liss B. How Parkinson's disease meets nucleolar stress. Biochim Biophys Acta. 2014; 1842:791–797. https://doi.org/10.1016/j.bbadis.2013.12.014 PMID: 24412806

52. Zeng J, Libien J, Shaik F, Wolk J, Hernández AI. Nucleolar PARP-1 Expression Is Decreased in Alzheimer's Disease: Consequences for Epigenetic Regulation of rDNA and Cognition. Neural Plasticity. 2016; 2016:8987928. https://doi.org/10.1155/2016/8987928 PMID: 27034851

53. Li D, Zhang J, Wang M, Li X, Gong H, Tang H, et al. Activity dependent LoNA regulates translation by coordinating rRNA transcription and methylation. Nature Communications. 2018; 9:1726–1739. https://doi.org/10.1038/s41467-018-04072-4 PMID: 29712923
